# Effectiveness of conventional versus virtual reality-based vestibular rehabilitation exercises in elderly patients with dizziness: a randomized controlled study with 6-month follow-up

**DOI:** 10.1016/j.bjorl.2021.08.010

**Published:** 2021-10-26

**Authors:** Tuba Kanyılmaz, Oya Topuz, Fazıl Necdet Ardıç, Hakan Alkan, Saadet Nur Sena Öztekin, Bülent Topuz, Füsun Ardıç

**Affiliations:** a Pamukkale University, Faculty of Medicine, Department of Physical Medicine and Rehabilitation, Denizli, Turkey; b Pamukkale University, Faculty of Medicine, Department of Otorhinolaryngology, Head and Neck Surgery, Denizli, Turkey

**Keywords:** Virtual reality, Vestibular rehabilitation, Dizziness, Elderly

## Abstract

**Objectives:**

To investigate the effect of vestibular rehabilitation exercises supported with virtual reality containing real-life environments on dizziness, static and dynamic balance, functional mobility, fear of falling, anxiety, and depression in elderly patients with dizziness.

**Methods:**

In this prospective randomized controlled study, 32-patients aged 65-years and older who applied to the otorhinolaryngology clinic with dizziness complaint randomly assigned to 2 groups. In Group 1 (n = 16), vestibular rehabilitation program, supported with virtual reality, and in Group 2 (n = 16), conventional vestibular rehabilitation program was applied 30-min a day, 5 sessions per week, 15 sessions in total for 3 weeks. Subjects were evaluated with The Vertigo Symptom Scale (VSS), Dizziness Handicap Inventory (DHI), Berg Balance Test (BBT) and Timed Up & Go Test (TUG), Falls Efficacy Scale-International (FES-I), Postural Stability Test (PST), Geriatric Depression Scale (GDS), Hamilton Anxiety Scale (HAS) at baseline, at the end of the treatment and 6-months after the treatment.

**Results:**

Statistically significant improvements were seen in the DHI emotional subscale and TUG in Group 1 compared to Group 2 at the end of the treatment in elderly with dizziness (*p* < 0.05). Also, there were significant improvements in the VSS, all DHI subgroups, and total scores, BBT, HAS in Group 1 compared to Group 2 at the 6-months after the treatment (*p* < 0.05).

**Conclusion:**

The application of vestibular rehabilitation in a virtual reality environment can lead to additional improvements especially in dizziness symptoms, disability, balance, and mobility in the elderly with chronic dizziness.

## Highlights

• Vestibular rehabilitation supported with virtual reality appears to be effective in elderly patients with dizziness.
• Virtual reality-based vestibular rehabilitation is superior to conventional vestibular rehabilitation therapy alone.
• The implementation of the virtual reality environment in the vestibular rehabilitation program may be useful.

## Introduction

Dizziness is a condition that includes disorders of spatial orientation and motion perception and may affect gaze stability, posture, and gait. This problem occurs approximately in 30% of the elderly in the community and its prevalence increases with age.^1^ Patient with a history of dizziness have limitations in daily activities, associated with increased fear of falling and fall-risk.^2^, ^3^

It has been shown that vestibular rehabilitation programs including postural control and balance exercises have positive effects on dizziness, balance, and risk of falling in the elderly.^4^, ^5^ Exercise programs are often performed in a clinical setting or at home. It has been reported that interactive video games and virtual reality provide new platforms for the delivery of exercise programs that can be used at home and for some users may be more enjoyable than typical exercise.^6^ Positive effects on walking, postural control, balance, and mobility in the elderly have been reported with exercises in the form of interactive games where virtual reality technologies are used.^7^, ^8^, ^9^, ^10^, ^11^ Recently a study showed that being exposed to immersive virtual reality using a head-mounted display is a feasible, safe approach to provide beneficial experiences to older adults with mobility, sensory, and/or cognitive impairments.^12^ Exercise programs containing real-life environments such as streets, supermarkets, parks, workplaces, where traffic is available can make a difference in patient motivation and exercise effectiveness. Although the suggestibility of virtual-reality in balance training in patients with vestibular disorders has already been stated, there have been conflicting data on the optimal conditions to perform virtual reality-based vestibular rehabilitation therapy. Furthermore, to the best of our knowledge, there is no study investigating the effects of virtual reality containing real-life environments created by 3D real-life videos played by virtual reality glasses and smartphone technology.

The objective of this study was to investigate the effect of vestibular rehabilitation exercises supported with virtual reality containing real-life environments on dizziness, static and dynamic balance, functional mobility, fear of falling, anxiety, and depression in elderly patients with dizziness.

## Methods

### Study design and participants

This prospective, randomized, single-blind, single-center, controlled study was conducted at Pamukkale University Faculty of Medicine between September 2017 to July 2018. A total of 91-patients aged 65-years and older who were admitted to the outpatient clinic of the otorhinolaryngology department with dizziness complaints were screened for eligibility. After a detailed history and physical examination including a detailed ear, nose, and throat examination, outpatient hearing, and other audiological tests along with vestibular tests including positional, oculomotor and postural tests had done. When necessary, radiological examinations including magnetic resonance imaging was requested from the patients in terms of differential diagnosis. Patients with uncompensated peripheral disease for more than 3-months were referred to the outpatient clinic of physical medicine and rehabilitation department for balance rehabilitation. The inclusion criteria were referring an otolaryngologist with the complaint of dizziness, living in society, and being able to stand up and walk by themselves without needing an assistive device. The criteria for exclusion were as follows; the presence of cognitive impairments (Mini-Mental State Examination score ≤ 23 points), presence of musculoskeletal or systemic disease that prevents exercise, participating in an exercise program in the last 6-months, presence of psychiatric or neurological diseases that affect cooperation and cognitive functions in their medical history, presence of neurological disease that affects balance in their medical history, presence of visual impairment, having positive Dix-Hallpike test and having lower 25 (OH)Vit-D level than 30 ng/mL. The study was prospectively registered at Clinical Trials. gov (NCT03412708). The study was designed in accordance with the Helsinki Declaration and the Institutional Review Board of Pamukkale University approved the study protocol (registration nº 60116787-020/63464, date of approval; September-19, 2017). Also, the subjects gave their written informed consent to participate in the study.

Patients who meet the inclusion criteria after initial screening and do not have exclusion criteria randomly assigned by layered block randomization into two groups, including sixteen patients in each group as follows: Group 1 received supervised vestibular rehabilitation supported with virtual-reality (n = 16), Group 2 received supervised vestibular rehabilitation program without virtual-reality (n = 16).

### Intervention

All patients were trained initially by a physician for 30 min, including a definition of fall, definition of fall prevention, risk factors, information on fall prevention, and recommendations for preventing falls.

The 3D videos consist of 2-media recorded with a 360 camera (Samsung Gear-360). (1) Video recorded in a broad square with heavy pedestrian and car traffic recorded with the real environmental noise, (2) Video recorded while walking among the aisles of a big supermarket where all shelves are full of goods in different shapes and colors. 3D videos were played with a smartphone (Samsung Galaxy-S7) attached to a virtual reality goggle (Samsung Gear VR-SM323). The duration of the videos was 15-min and 1.5-min, respectively. When the patients were wearing goggles, they could only see the playing 3D video. Patients in Group 1 performed the exercises with the goggles on and the video was playing in a clinical setting supervised by a physician. Exercises were conducted while sitting and standing in the 1^st^ video, and on the treadmill in the 2nd video.

Patients in Group 2 performed the exercises in a clinical setting supervised by the same physician without virtual reality.

Vestibular exercises were applied for three weeks, 5-times per week, 2-sets of 15-min, with a 5-minute break between sets, for a total of 35-min in both groups. Patients who did not attend at least 3-sessions during the intervention program dropped from the study. Compliance during the rehabilitation program was excellent as only one patient in each group missed on average 2/15 sessions. The patients were informed about the purpose of the exercises. During the exercises, assisted ambulation system (Biodex-Free-Step-SAS) was used to ensure the safety of the patient and to prevent falls. The exercise program consisted of exercise for eye movements, gaze stability, and postural stability was applied by subjects in the study groups. The exercise program was summarized in Table 1.

**Table 1:** Exercise program applied by subjects in the study groups.

Vestibular exercise program
**Exercises for eye movements**
*Smooth- Pursuit Eye Movements*: The patient is asked to move their eyes at first slowly and then quickly in horizontal and vertical directions (10-times). In the 1^st^ week while sitting, in the 2^nd^ week while standing, and in the 3^rd^ week while standing on soft ground.
*Saccadic eye movements*: The patient is asked to focus by moving their eyes 1-time per second to 2-targets 30 cm away from each other (10-times). Motion is applied in horizontal and vertical directions. While sitting on the 1^st^ week, standing on the 2^nd^ week, and standing on soft ground for the 3^rd^ week.
**Exercises for gaze stability**
*Training of Vestibulo-ocular reflex*: 1) The patient is asked to move their headfirst in horizontal, then in vertical directions for 1-minute, focusing on a fixed object. While sitting on the 1^st^ week, standing on the 2^nd^ week, and standing on soft ground for the 3^rd^ week. 2) The patient is asked to move their headfirst in horizontal, then in vertical directions for 1-minute, focusing on a moving object. While sitting on the 1^st^ week, standing on the 2^nd^ week, and standing on soft ground for the 3rd week.
*Training of Cervico-ocular reflex*: The patient sitting on a rotating chair is asked to move their torso to the right and left, keeping his head steady by focusing on a fixed object (10-times). It is conducted in the 1^st^ and 2^nd^ weeks.
**Exercises for postural stability**
*Standing up*: 1) Week: Bend forward and side to side while sitting, pass from sitting position to standing position and back to sitting position (10-times), standing in various positions (feet together, tandem position) (15 s), marching in place (10-times) is requested. 2) Week: Bending their body front and back, and to their sides (10 front and back, 10 to the sides) while standing, standing in various positions (feet together, tandem position) (15 s), marching in place (10-times) is requested. 3) Week: The exercises in the 2^nd^ week are conducted on soft ground.
*Walking*: Patients conduct their exercises while walking at a pace of 1.6 km/h on the treadmill. 1)Week: They are asked to walk forward on the treadmill (1-min). 2) Week: They are asked to walk on the treadmill while moving their head left-right, front-back (1-min). 3) Week: They are asked to walk on the treadmill while focusing on a fixed object and moving their heads first horizontally and then vertically (1-min)

### Assessment parameters

#### Vestibular questionnaire data

To assess the dizziness symptoms severity subjects filled out the Short form Vertigo Symptom Scale (VSS) which is a 15-item self-report rating questionnaire evaluating the frequency of dizziness and/or unbalance and accompanying autonomic and anxiety symptoms within the last one month.^13^, ^14^ The total score is between 0 and 60, higher scores indicate a more serious problem. A total score of 12 or more indicates severe dizziness. The validity and reliability of the Turkish version of VSS were shown by Yanık et al.^15^

Dizziness Handicap Inventory (DHI) was used to assess disability in patients with dizziness. It is a questionnaire consisting of 25 questions about the physical, functional, and emotional state of the patient. The maximum score for the emotional and functional subgroups is 36, and the maximum score for the physical function subgroup is 28, with a maximum score of 100 in total. Higher scores indicate more disability.^16^ The Turkish version of the DHI was shown to be reliable and valid.^17^

#### Balance assessment

Clinical dynamic balance was evaluated by the Berg Balance Test (BBT). In BBT, the level of qualification for each item is scored from zero to four, where 0 means “can not do it”; and 4 means “can do it independently and safely”. The total maximum score is 56 and the higher scores show a better balance.^18^ Şahin et al. showed that the Turkish version of the BBT was a reliable and valid scale for assessing balance in elderly adults.^19^

Postural stability tests were measured by Dynamic Posturography Biodex Balance System (Biodex, Inc., Shirley, New York).^20^, ^21^ There is a balance platform of this system in relation to computer software that allows objective evaluation of the system, which can be tilted up to 20° at a 360° range of motion. The overall stability score refers to the balance ability of the person in general and high values indicate that the balance is impaired. After a 20-s trial test, each person was subjected to three 20-s tests. A 10-s rest period was provided between each test, and as a result, averages of these three tests were obtained.

Timed-Up&Go (TUG) test was used to assess functional mobility. In this test, the elapsed time is measured in seconds for the individual to get up from the sitting position, walk a 3 m distance and return.^22^ The standard chair with armrests, a tape, cone, or other clear marker indicating the end of the distance should be required and the person should wear walking shoes.

#### The Psychological impact of dizziness

International Falls Efficacy Scale (IFES) was used to assess fear of falling. The test consists of 16 questions that assess patients’ anxiety about the possibility of falls during their daily activities.^23^ Each question is scored between 0 to 4. It was shown that the Turkish version of the IFES was reliable and valid for the elderly population.^24^

Geriatric Depression Scale (GDS) was used to screen depression in the elderly population in this study. The scale consists of a total of thirty closed-ended questions.^25^ The answers are calculated as “1” point for each question if answered in a depressing way, and the sum of these points and the total score are calculated. The higher scores indicate depressive characteristics. The validity and reliability of the GDS have been proven by translating it into Turkish.^26^

Hamilton Anxiety Scale (HAS) which was developed by Hamilton, questions the anxiety severity, psychological and somatic symptoms. When each of the 14-items is assessed, a score of 0–4 is given, and the patients are evaluated based on the scores they receive in the overall total. The higher the score, the higher the severity of anxiety.^27^ The total score is between 0–56, below 17 is mild, between 18–24 is moderate and 25–30 is severe. Reliability and validity of the Turkish version of the HAS were conducted by Yazıcı and his colleagues.^28^

### Statistics

The minimum sample size was calculated as 15-patients per group based on 95% power and a two-sided 0.05 significance level (α = 0.05, β = 0.95) using the G-power 3.1.9.4 statistical program. The sample size capable of detecting a change for the BBT was estimated using the expected amount of change in BBT data obtained from a previous study (Group 1 BBT score amount of change: 1.74 vs. Group 2 BBT score: 5.06).^29^

All statistical analyses were performed using SPSS-version 22.0 for Windows (Statistical-Package for the Social Sciences Inc, Chicago, IL, USA). Descriptive statistics were used to describe demographic characteristics. The Shapiro Wilk test was used to analyze the normal distribution assumption of the data. Continuous variables were analyzed with the Mann-Whitney-*U* test, whereas categorical variables were analyzed with a chi-squared test to compare the significance of the differences between two groups. In all analyses, *p*-values < 0.05 were considered statistically significant.

## Results

### Before starting the rehabilitation

**Figure 1:**
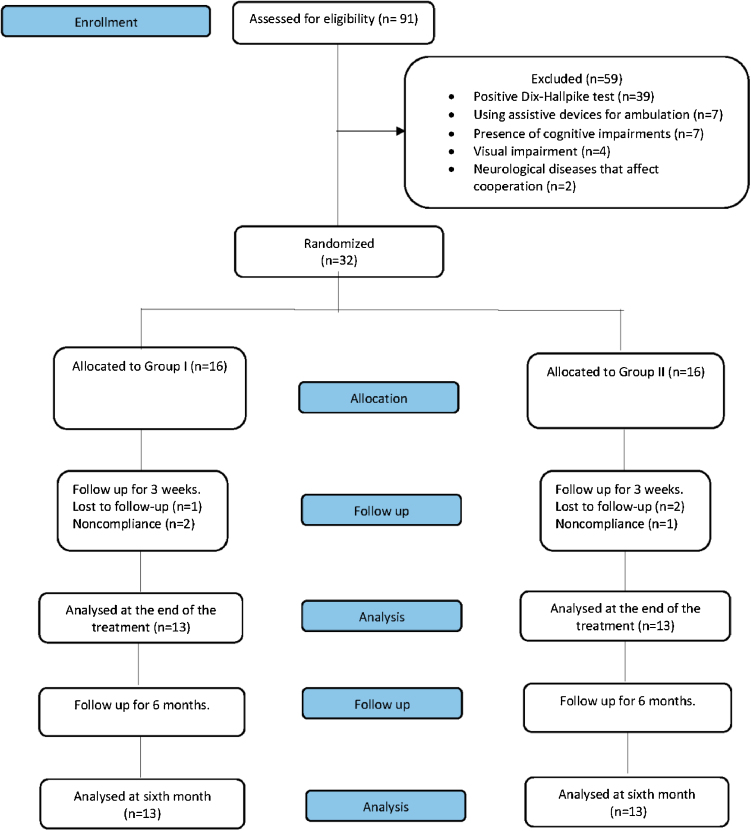
CONSORT flow diagram.

A total of 91 elderly patients with dizziness were screened for eligibility for this study, 59 of whom had to be excluded from the study; 39 of them due to the positive Dix-Hallpike test, 7 of them due to using assistive devices for ambulation, 7 of them due to presence of cognitive impairments, 4 of them due to the presence of visual impairment, 2 of them due to presence of neurological diseases that affect cooperation in their history. Two patients in Group 1 and one patient in Group 2 failed to complete the program because of non-compliance with the training schedule. Also, a patient in Group 1 and two patients in Group 2 failed to follow up. Therefore, 26 patients with dizziness of whom 10 were male and 16 were female, with a median age of 70-years were completed the entire study protocol (Fig. 1). There were no significant differences between the groups at baseline in terms of demographic and clinical characteristics (*p* > 0.05) (Table 2).

**Table 2:** Demographic and clinical characteristics of patients.

	Group 1 (n = 13)	Group 2 (n = 13)	*p*
**Age (years) median (IQR)**	70.00 (6.00)	70.00 (5.00)	0.500
**Gender, n (%)**			0.420
Female	7 (53.8)	9(69.2)	
Male	6 (46.2)	4 (30.8)	
**BMI (kg/m²) median (IQR)**	28.40 (5.20)	29.40 (5.70)	0.663
**Chronic disease, n (%)**			0.211
None	6 (46.2)	2 (15.4)	
One	3 (23.1)	6 (46.2)	
More than one	4 (30.8)	5 (38.5)	
**Smoker, n (%)**			0.308
Current smoker		1 (7.7)	
Never	13 (100)	12 (92.3)	
**Alcohol, n (%)**	–		0.308
User	1 (7.7)	–	
Not user	12 (92.3)	13 (100)	
**Medication, n (%)**			0.412
None	5 (38.5)	2 (15.4)	
Less than three	6 (6.2)	8 (61.5)	
More than three	2 (15.4)	3 (23.1)	
**Mini-Mental State Test, Median (IQR)**	28.00 (1.5)	27.00 (2.00)	0.179
**Lower extremity muscle strength, Median (IQR)**	14.00 (3.00)	12.00 (4.00)	0.778
**Lower extremity sensory examination, n (%)**			0.277
Normal	12 (92.3)	10 (76.9)	
Abnormal	1 (7.7)	3(23.1)	
**Eye movements, n (%)**			1.00
Normal	13 (100)	13 (100)	
Abnormal	–	–	
**Orthostatic hypotension, n (%)**			0.658
Present	3 (23.1)	4 (30.8)	
Absent	10 (76.9)	9 (69.2)	
**Cerebellar test, n (%)**			
Normal	13 (100)	13 (100)	
Abnormal	–	–	1.00
**Fall history, n (%)**			0.543
None	10 (76.9)	8 (61.5)	
One,	1 (7.7)	3 (23.1)	
More than one	2 (15.4)	2 (15.4)	
**Fracture history n (%)**			0.308
Present	–	1 (7.7)	
Absent	13 (100)	12 (92.3)	

Group 1, Vestibular rehabilitation supported with virtual reality; Group 2, Vestibular rehabilitation program without virtual reality; BMI, Body Mass Index.

### Immediately after rehabilitation

Statistically significant differences were found between two groups in DHI emotional subscale and TUG at the end of the treatment (*p* < 0.05) (Table 3, Table 4).

**Table 3:** Comparison of vestibular variables.

	Group 1	Group 2	p
	Median (IQR)	Median (IQR)	
**VSS**			
PreT	9.00 (11.00)	15.00 (18.00)	0.258
PostT	4.00 (6.50)	11.00 (18.00)	0.257
PostT6M	4.00 (10.00)	10.00 (11.50)	0.025
**DHI Functional**			
PreT	16.00 (12.00)	24.00 (16.00)	0.236
PostT	8.00 (14.00)	20.00 (18.00)	0.188
PostT6M	2.00 (12.50)	22.00 (14.50)	0.032
**DHI Emotional**			
PreT	14.00 (12.00)	8.00 (19.00)	0.918
PostT	4.00 (6.00)	10.00 (15.00)	0.046
PostT6M	0.00 (7.00)	8.00 (18.00)	0.049
PreT	14.00 (12.00)	8.00 (19.00)	0.918
**DHI Physical**			
PreT	18.00 (17.00)	12.00 (12.00)	0.661
PostT	6.00 (11.00)	12.00 (15.00)	0.353
PostT6M	4.00 (12.00)	14.00 (11.00)	0.047
**DHI Total**			
PreT	50.00 (37.00)	48.00 (43.00)	0.625
PostT	18.00 (28.00)	42.00 (38.00)	0.117
PostT6M	8.00 (25.00)	44.00 (35.00)	0.015

Group 1, Vestibular rehabilitation supported with virtual reality; Group 2, Vestibular rehabilitation program without virtual reality; *p*, Mann-Whitney U test; PreT, Pretreatment; PostT, Posttreatment; PostT6M, 6-month Posttreatment; VSS, Vertigo Symptom Scale; DHI, Dizziness Handicap Inventory.

**Table 4:** Comparison of balance variables.

	Group 1	Group 2	*p*
	Median (IQR)	Median (IQR)	
**BBT**			
PreT	50.00 (6.00)	51.00 (6.00)	0.737
PostT	54.00 (3.00)	52.00 (5.00)	0.108
PostT6M	54.00 (4.00)	51.00 (5.00)	0.042
**PST-GSI**			
PreT	1.20 (0.55)	1.30 (0.85)	0.604
PostT	1.00 (0.75)	0.80 (0.50)	0.777
PostT6M	1.00 (0.45)	1.00 (0.40)	0.979
**PST-APSI**			
PreT	0.90 (0.55)	0.80 (0.45)	0.661
PostT	0.50 (0.55)	0.60 (0.50)	0.470
PostT6M	0.70 (0.35)	0.70 (0.40)	0.757
**PST-MLSI**			
PreT	0.70 (0.40)	0.80 (0.50)	0.502
PostT	0.50 (0.50)	0.50 (0.30)	0.816
PostT6M	0.50 (0.35)	0.40 (0.40)	0.897
**FRI**			
PreT	1.10 (0.50)	1.20 (0.45)	0.439
PostT	0.80 (0.80)	0.70 (0.40)	0.643
PostT6M	0.80 (0.55)	1.00 (0.35)	0.222
**TUG**			
PreT	14.00 (2.50)	15.00 (5.00)	0.286
PostT	11.00 (1.50)	13.00 (4.50)	0.004
PostT6M	13.00 (4.50)	14.00 (6.00)	0.368

Group 1, Vestibular rehabilitation supported with virtual reality; Group 2, Vestibular rehabilitation program without virtual reality; p, Mann-Whitney U test; PreT, Pretreatment; PostT, Posttreatment; PostT6M, 6-month posttreatment; BBT, Berg Balance Test; GSI, General Stability Index; MLSI, Medio-lateral Stability Index; APSI, Anteroposterior Stability Index; FRI, Falling Risks Index; TUG, Timed Up&Go test.

### At the end of 6-months after rehabilitation

There were significantly greater improvements in the VSS, all DHI-subgroups, and total scores, BBT, HAS in Group 1 compared to Group 2 at the 6-months after the treatment (*p* < 0.05) (Table 3, Table 4, Table 5). On the other hand, there were no statistically significant differences between groups in terms of postural stability, fear of falling, and depression at the 6-months after the treatment (*p* > 0.05). There were no adverse events attributable to the study protocol.

**Table 5:** Comparison of the psychological variables.

	Group 1	Group 2	*p*
	Median (IQR)	Median (IQR)	
**IFES**			
PreT	25.00 (18.00)	25.00 (19.00)	0.778
PostT	19.00 (7.00)	26.00 (11.00)	0.123
PostT6M	21.00 (10.50)	26.00 (17.00)	0.205
**GDS**			
PreT	7.00 (11.50)	8.00 (8.00)	0.328
PostT	6.00 (6.50)	9.00 (8.00)	0.246
PostT6M	7.00 (9.00)	12.00 (11.00)	0.302
***HAS***			
PreT	3.00 (6.00)	6.00 (4.00)	0.551
PostT	1.00 (3.50)	2.00 (8.50)	0.176
PostT6M	1.00 (2.00)	5.00 (6.50)	0.008

Group 1, Vestibular rehabilitation supported with virtual reality; Group 2, Vestibular rehabilitation program without virtual reality; p, Mann-Whitney U test; PreT, Pretreatment; PostT, Posttreatment; PostT6M, 6-month posttreatment; IFES, International Falls Efficacy Scale; GDS, Geriatric Depression Scale; HAS, Hamilton Anxiety Assessment Scale.

## Discussion

This randomized controlled study was the first to investigate the effect of vestibular rehabilitation supported by 3D-real-life videos played on virtual-reality glasses and smartphone technology in elderly patients with dizziness in the short- and long-term follow-up. Our results revealed that the virtual reality-based vestibular rehabilitation exercises may be an effective treatment in reducing dizziness, disability due to dizziness, balance, functional mobility, and anxiety in elderly patients with dizziness.

The use of virtual reality technology for vestibular rehabilitation can induce retinal slip and promote adaptation and compensation. The induction of retinal slip creates optokinetic eye movements, which stimulate adaptation mechanisms.^30^, ^31^ In a recent study, it was used a headset and phone displaying a rollercoaster ride induce a visual and vestibular challenge to postural stability in 28 healthy, physically active people. The researchers stated that the virtual-reality environment applied by smartphones creates difficulties on postural stability control and can be used as a training stimulus.^32^ Furthermore, it was reported that exposure to the virtual environment with a head-mounted virtual reality system may have the potential to produce adaptive changes in the central vestibular system by creating a temporary reduction in Vestibulo-Ocular Reflex (VOR) gain.^33^ In a preliminary study, subjects performed a search task that included active head movements on a 360-degree visual display, while the control group applied the same adaptation protocol without using a virtual screen. At the end of the week, researchers found an increase in VOR gain and improvement in DHI in subjects using visual screens. Researchers concluded that immersive computing environments can increase VOR gain and reduce vertigo.^34^ In our study, a more comprehensive vestibular rehabilitation program including static and dynamic postural stability exercises was applied together with gaze stabilization exercises including active head and eye movements. At the end of the 3-week treatment, the effect of exercise in the virtual environment using virtual glasses and smartphone technology on the improvement of the emotional score of DHI was superior to the control group.

Particularly, evidence is scarce regarding the efficacy of using virtual reality in the elderly. Unlike our study, it was reported that virtual reality-based treatment produces equivalent effects on balance, dizziness-related disability, functional mobility, gait, and anxiety in patients with the vestibular disorder compared with clinically accepted customized vestibular rehabilitation including gaze stabilization, standing balance, and walking exercises. In the virtual reality-based treatment group, they walked on a treadmill in an immersive virtual market environment.^35^ In our study, patients were over 65-years of age and had only peripheral vestibular insufficiency unlike in that study. Also, we found that using virtual reality in addition to vestibular rehabilitation was significantly superior to vestibular rehabilitation therapy alone on the DHI emotional subscale and TUG. Furthermore, the cumulative treatment time in the virtual environment was also longer in our study. It has been reported that the magnitude of the effect of treatment in virtual-reality treatments is associated with cumulative exposure to the virtual-reality environment.^36^ Additionally, it was stated that older adults’ tendency to incorporate spatially conflicting and unreliable visual cues into their self-motion percept may affect their performance on mobility-related tasks like walking and driving. Vestibular rehabilitation in a virtual-reality environment may have different effects on the elderly and young people.^37^

Virtual reality settings are extremely useful for vestibular disorders and strongly recommended. Recent randomized controlled trials compared the use of a home exercise program including only traditional vestibular rehabilitation (control-group) and the Track Speed Racing 3D-game with a head-mounted display and smartphone (experimental-group) in patients with chronic unilateral vestibular hypofunction.^38^, ^39^ Micarelli et al. found that the improvement in VOR gain on the lesional side, in posturography parameters, as well as in DHI and Activities-specific Balance Confidence scale scores of the experimental group, was significantly greater than that of the control group in the short-term (4-week).^38^ Viziano et al. then reported that the significant difference between the two groups remained in the long-term (56-week) follow-up, even though both groups discontinued their home exercise program after the first 4-weeks.^39^ In our study, the virtual reality-based vestibular rehabilitation did not provide more improvements in the posturographic scores, DHI total, and subscale scores except the emotional subscale at the end of the 3-weeks treatment. Unlike the study of Micarelli et al., exercises were performed in a virtual environment instead of a game procedure in the experimental group. Also, the shorter duration of treatment, the lower number of patients, and the older age in our patient population may have resulted in fewer outcome improvements in the short-term. However, similarly to the study of Viziano et al., we showed that the virtual reality-based vestibular rehabilitation provided more improvements in the VSS, DHI all-subgroups, and total score, BBT, HAS at the 6-month follow-up. In long-term, the implementation of the virtual-reality environment in the vestibular rehabilitation program may be useful by more adapting to real-life environments such as a street, supermarket, park, workplace, where traffic is available, and spending more time in these environments in elderly patients with dizziness.

This study is that is the first prospective randomized-controlled study demonstrating the beneficial effect of virtual reality-based vestibular rehabilitation using virtual glasses and smartphone technology on dizziness, balance, and functional mobility in the elderly with dizziness. The limitation of this study is that the small number of patients may not be enough to draw strong conclusions on the clinical effects of virtual-reality-based vestibular rehabilitation. Further longitudinal, prospective studies are warranted to evaluate the long-term results of the virtual-reality-based vestibular rehabilitation, using a larger sample size with a long-term follow-up.

## Conclusion

Vestibular rehabilitation supported with virtual reality may have additional effects in elderly patients with dizziness. The application of vestibular rehabilitation in a virtual reality environment can lead to additional improvements especially in dizziness symptoms, disability, balance, and mobility in the elderly with chronic dizziness.

## Funding

This study is funded by Pamukkale University Scientific Research Fund with Project nº 2016TIPF025.

## Ethics approval

The Institutional Review Board of Pamukkale University approved the study (registration nº 60116787-020/63464, date of approval; September 19, 2017), and the subjects gave their written informed consent to participate in the study.

## Trial registration

The study was prospectively registered at Clinical Trials. gov (NCT03412708).

## Level of evidence

1A: Prospective randomized-controlled study.

## Conflicts of interest

The authors declare no conflicts of interest.
